# Association between hypothyroidism, levothyroxine replacement and kidney function outcomes: a systematic review and meta-analysis

**DOI:** 10.3389/fendo.2026.1841255

**Published:** 2026-05-01

**Authors:** Xichang Wang, Xueying Shang, Yang Li, Cihang Lu, Ying Shao, Dongyu Yang, Weiping Teng, Xiaoguang Shi

**Affiliations:** 1 Department of Endocrinology, Shengjing Hospital of China Medical University, Shenyang, Liaoning, China; 2 Department of Endocrinology, The Fourth Affiliated Hospital of China Medical University, Shenyang, Liaoning, China; 3 Department of Pulmonary and Critical Care Medicine, Shengjing Hospital of China Medical Universitym, Shenyang, Liaoning, China; 4 Department of Endocrinology and Metabolism and the Institute of Endocrinology, National Health Commission Key Laboratory of Diagnosis and Treatment of Thyroid Diseases, The First Hospital of China Medical University, Shenyang, Liaoning, China

**Keywords:** chronic kidney disease, glomerular filtration rate, hypothyroidism, levothyroxine, thyroid function

## Abstract

**Objective:**

Regarding the exact association between hypothyroidism, levothyroxine replacement and kidney dysfunction, there is a lack of study to summarize the previous evidence. The aim of this study is to explore these relationships.

**Methods:**

The PubMed, Cochrane Library, EMBASE, and Google Scholar were searched for observational studies and RCTs. The included studies were conducted in adult nonpregnant participants and analyzed kidney outcomes of interest in serologically confirmed hypothyroidism patients. Additionally, studies that examined kidney outcomes during levothyroxine treatment were retrieved. Standard Mean Difference (SMD), Odds Ratios (ORs) and Risk Ratios (RRs) were estimated as the effect variables.

**Results:**

Totally, 46 studies were included. Both non-distinct hypothyroidism and subclinical hypothyroidism (SHypo) were significantly associated with the prevalence of chronic kidney disease (CKD) (OR 1.94, 95% CI [1.62, 2.32]) (OR 1.87, 95% CI [1.55, 2.27]). Non-distinct hypothyroidism and SHypo were also associated with estimated glomerular filtration rate (eGFR) reduction (SMD -0.68, 95% CI [-0.81, -0.55]) (SMD -0.99, 95% CI [-1.59, -0.38]). No significant association between hypothyroidism and CKD incidence was found, and its association with end-stage renal disease (ESRD) was not significant in patients with underlying kidney dysfunction. No significant improvement in eGFR was observed after levothyroxine treatment, either in non-distinct hypothyroidism or in SHypo patients.

**Conclusion:**

This study verifies a cross-sectional association between hypothyroidism and kidney dysfunction. However, no definitive prospective association was found. The bidirectional causal association between hypothyroidism, levothyroxine replacement and kidney outcomes remain controversial based on evidence from extensive observational studies. More large-scale randomized controlled trials are needed to supplement this evidence in practice to avoid overtreatment or undertreatment.

**Response:**

The registraton information has been mentioned in the Methods section.

## Introduction

Chronic kidney disease (CKD) is defined as abnormalities in kidney structure or function persisting for at least three months with implications for health, which increase the risks of comorbidities such as cardiovascular events, anemia and electrolyte disturbance (1). According to population studies in different countries, the prevalence of CKD ranges from 7.2% to 13.4% (2–6). Due to its high prevalence and mortality rate, timely diagnosis and treatment of CKD is essential. Deep concern has been raised about the reduced survival rate in CKD patients, and efforts to identify comorbidities that could delay its progression are of paramount significance. There is still controversy regarding which specific population should undergo CKD screening. Among those risk factors for CKD, the role played by thyroid dysfunction requires further summarization and investigation. Several efforts have been undertaken to demonstrate the importance of screening at-risk patients; however, gaps still need to be filled. Given the vital role of the thyroid in regulating the renal system, it is worthwhile to investigate whether hypothyroidism can contribute to the incidence and deterioration of kidney function. A nationwide survey by our research team revealed that the prevalence of hypothyroidism exceeds 13% in mainland China, with the majority of patients having subclinical hypothyroidism (SHypo) (7). Moreover, the upper limit of the thyroid-stimulating hormone (TSH) reference interval was 7.04 mU/L in the reference population, which is much higher than the corresponding value of the Roche kit (8). Given the significant effects of metabolic factors on CKD and the widespread effects of hyperthyrotropinemia, the relationship between them should be systematically assessed.

In clinical practice, patients with severe overt hypothyroidism are at high risk of kidney dysfunction. Two Mendelian randomization studies have demonstrated a significant causal association between hypothyroidism and CKD (9, 10). This association has also been reported in previous high-quality observational studies (11–13). However, some confusion remains in this field. First, the associations between SHypo and kidney outcomes require further confirmation. Second, it is currently unclear what effect levothyroxine (LT4) has on kidney-related clinical outcomes in hypothyroidism patients. Third, differences in participants’ age, gender, race, etc., can lead to inconsistent findings in previous studies. Fourth, the reference intervals for thyroid function and parameters or formulas for kidney function were inconsistent across the studies. Studies on the above topic have been continuously reported over the past two decades. However, a systematic review and meta-analysis summarizing their findings is lacking.

The purpose of this study was to comprehensively elucidate the associations between hypothyroidism (especially SHypo) and kidney outcomes and to explore the changes in kidney outcomes after LT4 replacement in hypothyroidism patients.

## Methods

The protocol for this study was registered with PROSPERO (CRD420251114388). Data for this study were extracted from published papers, thus ethical review was exempted by our institution. The preferred reporting items for systematic reviews and meta-analyses (PRISMA) reporting guidelines were followed (**Supplementary Table 1**) (14).

### Literature search strategy

The PubMed, Cochrane Library, EMBASE, and Google Scholar databases were searched for observational studies and randomized clinical trials (RCTs). A total of three researchers were responsible for the literature search. The retrieval formula was as follows: ((renal function [All Fields]) OR (kidney function [All Fields])) AND (((((hypothyroidism [All Fields]) OR (hypothyroid [All Fields])) OR (LT4 [All Fields])) OR (L-thyroxine [All Fields])) OR (levothyroxine)). The three researchers sent the records to a fourth researcher, who then screened out duplicate records. Additionally, another researcher was tasked with scanning references in review articles to collect records that were not yet discovered by the above researchers. Through the above process, we initially included a total of 601 studies.

### Inclusion and exclusion criteria for the studies

The inclusion criteria for the studies are described below. First, the clinical outcomes included the crude prevalence or incidence of CKD, or one or more biochemical tests reflecting kidney function. Second, the number of cases or incidence events of CKD and the means and SDs of continuous variables were available. Third, the confirmation of hypothyroidism was newly-diagnosed and based on laboratory measurements rather than personal history collection. Fourth, the etiology of hypothyroidism must be primary, not central or iatrogenic, to minimize the influence of other types of thyroid diseases or irrelevant treatments on kidney function. Fifth, the researchers explicitly reported that the serum TSH levels returned to the euthyroid range after LT4 replacement.

**Figure 1:**
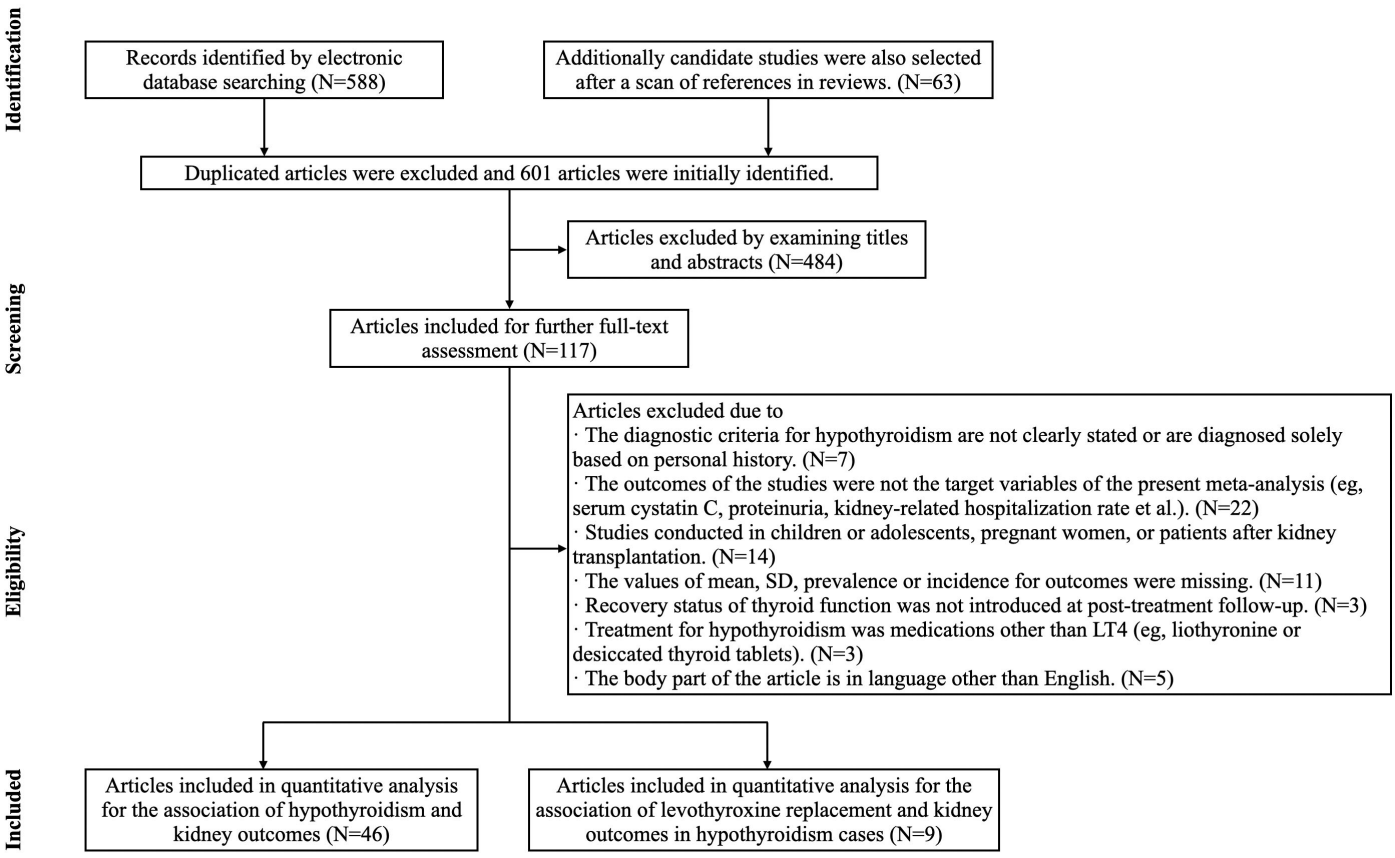
PRISMA flow diagram of study selection.

Studies were excluded if they met any of the following criteria. First, the reference interval of serum thyroid function was not clearly reported. Second, studies involving children or adolescents, pregnant women, or patients after kidney transplantation were excluded. Third, for the analysis of the effects of LT4 replacement on kidney outcomes, studies were excluded if the patients had other types of thyroid diseases or received thyroid hormone treatment other than LT4. Fourth, if there was an overlap in the populations between two candidate studies, the study with the smaller sample size was excluded. Fifth, several kidney outcomes that were not of interest, such as serum cystatin C levels, the proteinuria prevalence, kidney-related hospitalization rates, etc., were not considered because there was too little support from previous evidence. Finally, non-English literature, brief reports, letters, basic experiments, conference submissions, etc., were also excluded. The screening criteria are shown in **Figure 1**.

### Quality assessment

The Newcastle–Ottawa Scale was selected for quality evaluation (15). The scale for case–control studies was applied when kidney outcomes in participants with different thyroid function statuses were compared. The scale used in cohort studies was referred to when analyzing the effects of LT4 replacement on kidney outcomes in patients with hypothyroidism. Briefly, the scale for case–control studies include the following three items: selection of individuals, comparability between the hypothyroidism patients and the non-hypothyroidism controls, and confirmation of the diagnosis of hypothyroidism. The scale for cohort studies includes the following three items: selection of hypothyroidism patients, intergroup comparability between hypothyroidism patients and control individuals, and selection and assessment of kidney outcomes. The maximum score for both scales is nine. Only studies with a score of five or above were considered for inclusion in this meta-analysis. After the screening process shown in **Figure 1**, all the candidate studies met the criteria and could be used for further analysis.

### Determination of variables

A preliminary literature search revealed a paucity of epidemiological studies examining the impact of overt hypothyroidism on kidney outcomes. In addition, given the well-established association between overt hypothyroidism and kidney dysfunction in practice, we did not consider patients with overt hypothyroidism as a distinct subgroup in the present study. The independent variables in this study were overall hypothyroidism without distinction, as well as SHypo.

Continuous variables reflecting kidney function in this study included the serum urea level, serum creatinine level, and estimated glomerular filtration rate (eGFR). The conversion factors for different units were consistent with the 2024 KDIGO guidelines (1). The Chronic Kidney Disease Epidemiology Collaboration (CKD-EPI) formula or the Modification of Diet in Renal Disease (MDRD) formula was used to calculate the eGFR. CKD was defined as an eGFR <60 mL/min/1.73 m^2^, corresponding to stage G3–5 CKD. End-stage renal disease (ESRD) was diagnosed on the basis of either the eGFR or treatment history. ESRD was defined as an eGFR <15 mL/min/1.73 m^2^ in most cases, corresponding to stage G5 CKD. ESRD was also confirmed if patients were described as having dialysis-dependent CKD.

### Statistical analysis

Two independent investigators extracted the means, SDs, and numbers of participants for each kidney outcome. For kidney outcomes presented as continuous variables, the inverse variance method was used to analyze the association between hypothyroidism or LT4 replacement and the outcome, and the standard mean difference (SMD) was regarded as the effect variable. For kidney outcomes presented as dichotomous variables, the Mantel–Haenszel method was used, and odds ratios (ORs) or risk ratios (RRs) were applied as the effect variables. The heterogeneity among the studies was estimated with the chi-square-based Q test and the I^2^ test; I^2^ ≤ 50% and I^2^ > 50% corresponded to a fixed-effects model and a random-effects model, respectively. Unless otherwise specified, sensitivity analysis was conducted by the sequential removal of the studies. The meta-analysis was performed by using Review Manager (Version 5.4.1; The Cochrane Collaboration, 2020). Publication bias was assessed by using Egger test plots in Stata 16 (Stata Corp, College Station, TX).

## Results

### Characteristics of the included studies

In total, 46 studies, covering more than 800,000 individuals, were included in the meta-analysis of the associations between hypothyroidism and kidney outcomes (11–13, 16–58). As shown in **Table 1**, the time span of the studies ranged from 1998 to 2025. Among these studies, 18 did not differentiate the severity of hypothyroidism, 18 merely analyzed the associations between SHypo and kidney outcomes, and nine studies differentiated the severity of hypothyroidism.

**Table 1:** General characteristics of included studies on the association between hypothyroidism and reduced kidney function.

Author	Published year	Country	No. subjects	Mean age	Gender composition (male/female)	Diagnostic criteria for hypothyroidism	Kidney outcomes
Lin	1998	China	356	ESRD patients, 57.0 ± 15.2; controls, 56.8 ± 14.9	201/155	TSH > 3.1 mU/L	Prevalence of ESRD
Lo	2005	US	14623	48.7 ± 18.9	6952/7671	TSH > 4.5 mU/L or treatment with thyroid hormone	Prevalence of CKD
Manetti	2005	Italy	181	SHypo, 37 ± 13; controls, 41 ± 15	42/139	SHypo, TSH 4.5–10 mU/L, normal free thyroid hormones	Serum creatinine
Chonchol	2008	Italy	3089	54.9 ± 16.2	682/2407	SHypo, TSH > 4.5 mU/L, normal FT4	Prevalence of CKD
Arora	2009	India	176	Hypothyroidism, 46.55 ± 1.8; controls, 45.72 ± 2.1	52/124	TSH > 6 mU/L	Serum creatinine
Asvold	2011	Norway	29480	Range: 41-98	9769/19711	SHypo, TSH 3.6-4.0 mU/L or TSH > 4.0 mU/L and FT4 ≥ 8.0 pmol/L; OHypo, TSH > 4.0 mU/L and FT4 < 8.0 pmol/L	Prevalence of CKD
Jusufovic	2011	Bosnia and Herzegovina	80	ESRD cases, 53.28 ± 11.76; controls, 49.43 ± 7.80	38/42	TSH > 4.4 mU/L	Prevalence of ESRD
Ng	2012	Taiwan	117	50.65 ± 11.26	45/72	SHypo, TSH > 4mU/L, normal FT4	Serum creatinine and urea
Saini	2012	India	244	Range: 20-50	N.A.	SHypo, TSH 6.1-9.9 mU/L with normal FT4 and FT3	Serum creatinine and urea
Kim	2013	Korea	168	64.8 ± 14.6	88/80	SHypo, TSH > 4.20 mU/L, normal FT4	eGFR
Ye	2013	China	8126	44.99 ± 12.05	8126/0	SHypo, TSH > 4.00 mU/L, normal FT4	Prevalence of CKD, eGFR
Drechsler	2014	Germany	1000	Range: 18-80	531/469	SHypo, TSH 4.1-15.0 mU/L, normal free thyroid hormones	Serum creatinine
Meuwese	2014	Netherlands	555	85	187/368	SHypo, TSH > 4.5 mU/L, normal FT4	eGFR
Jia	2015	China	933	SHypo, 63.68 ± 11.11; controls, 61.45 ± 12.29	437/496	SHypo, TSH > 5.00 mU/L, normal FT4 and FT3	Prevalence of CKD; Serum creatinine and urea; eGFR
Yuan	2015	China	22133	56.74 ± 8.62	7806/14327	TSH > 4.2 mU/L	Prevalence of CKD
Bajaj	2016	India	41	54.1 ± 14.6	N.A.	TSH > 4 mU/L	eGFR; Serum creatinine and urea
Chuang	2016	Taiwan	41454	75.8 ± 6.4	21531/19923	SHypo, TSH 5–10 mU/L; OHypo, TSH 10–99 mU/L	Incidence of CKD (median follow-up three years)
Costa	2016	Brazil	104	47 ± 12	41/63	TSH > 4.67 mU/L	Prevalence of ESRD
Fan	2016	China	279	67.8 ± 14.1	149/130	TSH > 5.6 mU/L	Prevalence ESRD
Miranda	2017	Brazil	13193	Median and IQR, 51 (45-58)	6353/6840	SHypo, TSH > 4.0 mU/L, normal FT4	Prevalence of CKD, eGFR value
Sanai	2017	Japan	37	57.9 ± 22.2	16/21	TSH ≥ 4.83 mU/L	Prevalence of ESRD
Pakfetrat	2017	Iran	172	ESRD patients, 57.2 ± 17.2; controls, 56.6 ± 16.8	96/76	TSH > 4.05 mU/L	Prevalence of ESRD
Schultheiss	2017	US	12785	57.4 ± 5.7	5561/7224	TSH over race-specific reference values (whites, 5.4 mU/L; blacks, 4.2 mU/L)	Prevalence of CKD, eGFR; incidence of CKD (median follow-up 19.6 years)
Sforza	2017	Argentina	451	77.0 ± 7.9	208/243	SHypo, TSH > 5 mU/L, normal TT4 and TT3; OHypo, TSH > 5 mU/L, TT4 < 4.5 μg/dL	Prevalence of CKD and ESRD
Chen	2018	China	126	SCH without treatment, 56 ± 4; SCH with LT4 treatment, 51 ± 5; controls, 49 ± 5	44/82	SHypo, TSH > 4.94 mU/L, with normal FT4 and FT3	Serum creatinine, eGFR
Patil	2018	India	608	Range: 18-70	228/380	SHypo, TSH > 5.5 mU/L with normal FT4 and FT3	Serum creatinine, eGFR
Rhee	2018	US	227426	71 ± 10	Female proportion, 3%	TSH > 5.0 mU/L	eGFR
Tanaka	2018	Japan	888	56.6 ± 9.1	351/537	SHypo, TSH 4.5-20.0 mU/L	eGFR
Zhang	2018	China	5936	62 ± 11	2909/3027	SHypo, TSH > 4.78 mU/L, normal FT4	Prevalence of CKD, eGFR
Alshammari	2019	Saudi Arabia	255	>18	160/95	OHypo, TSH > 5.5 mU/L with FT4 < 12 pmol/L; SHypo, TSH > 5.5 mU/L with normal FT4	Prevalence of ESRD
Toda	2019	Japan	16390	54.0 ± 11.2	11110/5280	TSH > 4.26 mU/L	Prevalence of CKD, eGFR; incidence of CKD (median follow-up three years)
Huang	2020	US	378101	67.0 ± 8.9	164002/214099	TSH > 4.00 mU/L and/or thyroid hormone treatment	Prevalence of CKD and ESRD, eGFR
Schultheiss	2020	Germany	4600	49.6 ± 18.4	2733/1867	TSH >3.29 mU/L and FT4 within the reference range or <9.8 pmol/L	eGFR
Torkian	2020	Iran	239	49.95 ± 16.19	117/122	SHypo, TSH > 4.0 mU/L, normal FT4	Serum urea
Zijlstra	2020	UK	4864	75.3 ± 3.4	2385/2479	SHypo, TSH >4.5 mU/L with normal FT4 level	Prevalence of CKD, serum urea
Kazempour-Ardebili	2021	Iran	5626	40.6 ± 14.3	2371/3255	SHypo, TSH > 5.06 mU/L with normal FT4; OHypo, TSH > 5.06 with FT4 < 11.71 pmol/L	Prevalence of CKD
Anum	2022	Pakistan	400	Range: 18-60	221/179	SHypo, TSH 5–10 mU/L, normal FT4 and FT3	Prevalence of CKD
Santivanez	2022	Spain	299	71 ± 13	184/115	SHypo, TSH > 5.3 mU/L, normal FT4	eGFR
Ansari	2023	India	200	>18	147/53	TSH > 4.8 mU/L	Prevalence of ESRD
Brenta	2023	Argentina	246	Median and IQR, 73 (68-77)	79/167	SHypo, TSH > 6 mU/L, normal FT4	Prevalence of CKD
Ittermann	2023	Germany	7933	Range: 20-93	3876/4057	TSH > 3.29 or 4.20 mU/L	Serum creatinine and urea, prevalence of CKD
Kim	2023	Korea	3257	44.10 ± 0.28	1762/1495	SHypo, TSH > 6.68 mU/L, normal FT4	Prevalence of CKD, eGFR
Matsuoka-Uchiyama	2023	Japan	553	60 ± 15	285/268	TSH > 4.20 mU/L	eGFR, serum creatinine
Shakya	2023	India	192	Male, 42 ± 18; female, 38 ± 11	124/68	SHypo, TSH 5.5–10 mU/L	eGFR
Hafed	2024	Saudi Arabia	200	63.40 ± 16.09	120/80	TSH > 5.5 mU/L	Prevalence of ESRD, eGFR, serum creatinine and urea
German	2025	Pakistan	131	56.4 ± 3.6	74/57	TSH > 5.6 mU/L	Serum creatinine and urea

The units of serum TSH may not be consistent across studies. All TSH units in the above table were converted to mU/L, which is more commonly used internationally, for comparison purposes and in combination with clinical practice. Even though the definition of CKD was not mentioned in some literature, we defined CKD as eGFR < 60 mL/min/1.73m^2^ to facilitate comparison across studies, also corresponding to Stage G3–5 CKD in some of the studies. The Ittermann et al. study consisted of two populations (SHIP study and BASE-II study), in which the TSH reference intervals were not consistent. ESRD, end stage renal disease; CKD, chronic kidney disease; eGFR, estimated glomerular filtration rate; TSH, thyroid stimulating hormone; FT4, free thyroxine; TT4, total thyroxine; SHypo, subclinical hypothyroidism; OHypo, overt hypothyroidism.

Additionally, nine studies examined changes in kidney function before and after LT4 replacement (**Table 2**) (20, 30, 39, 59–64). Changes in serum creatinine levels and the eGFR were also assessed. These studies were small-sample prospective studies with follow-up durations ranging from three months to three years.

**Table 2:** General characteristics of included studies on the analysis of kidney function changes in hypothyroidism patients before and after LT4 replacement.

Author	Published year	Country	No. subjects	Mean age	Diagnostic criteria for hypothyroidism	Initial LT4 dosage (μg/L)	Treatment duration	Kidney outcomes
Hollander	2005	Netherlands	37	47 ± 16	TSH > 4.0 mU/L	–	3 months or longer	Serum creatinine, eGFR
Arora	2009	India	46	46.55 ± 1.8	TSH > 6 mU/L	–	6 months	Serum creatinine
Goede	2009	Switzerland	16	44 ± 18	TSH > 4.2 mU/L	–	4 ± 2 months	Serum creatinine
Shin	2012	Korea	180	61.6 ± 12.4	SHypo, TSH 4.94–10 mU/L with normal FT4	25 μg/d	36 months	eGFR
Liu	2015	China	60	49 ± 10	SHypo, TSH 4.0-7.0 mU/L with normal FT4 and FT3	12.5 μg/d	48 weeks	Serum creatinine, eGFR
Bajaj	2016	India	9	48.4 ± 17.5	TSH > 4.0 mU/L	1.6 μg/kg	6 months	eGFR
Bajaj	2017	India	32	42.3 ± 16.8	TSH > 4.0 mU/L	1.6 μg/kg for OHypo and 12.5-50 μg/d for SHypo patients	6 months	eGFR
Chen	2018	China	43	51 ± 5	SHypo, TSH > 4.94 mU/L with normal FT4 and FT3	12.5 μg/d	24 weeks	Serum creatinine, eGFR
Naguib	2023	Egypt	41	38 ± 4	TSH > 4.20 mU/L	–	–	Serum creatinine, eGFR

### Associations between hypothyroidism and the prevalence of CKD and ESRD

**Figure 2:**
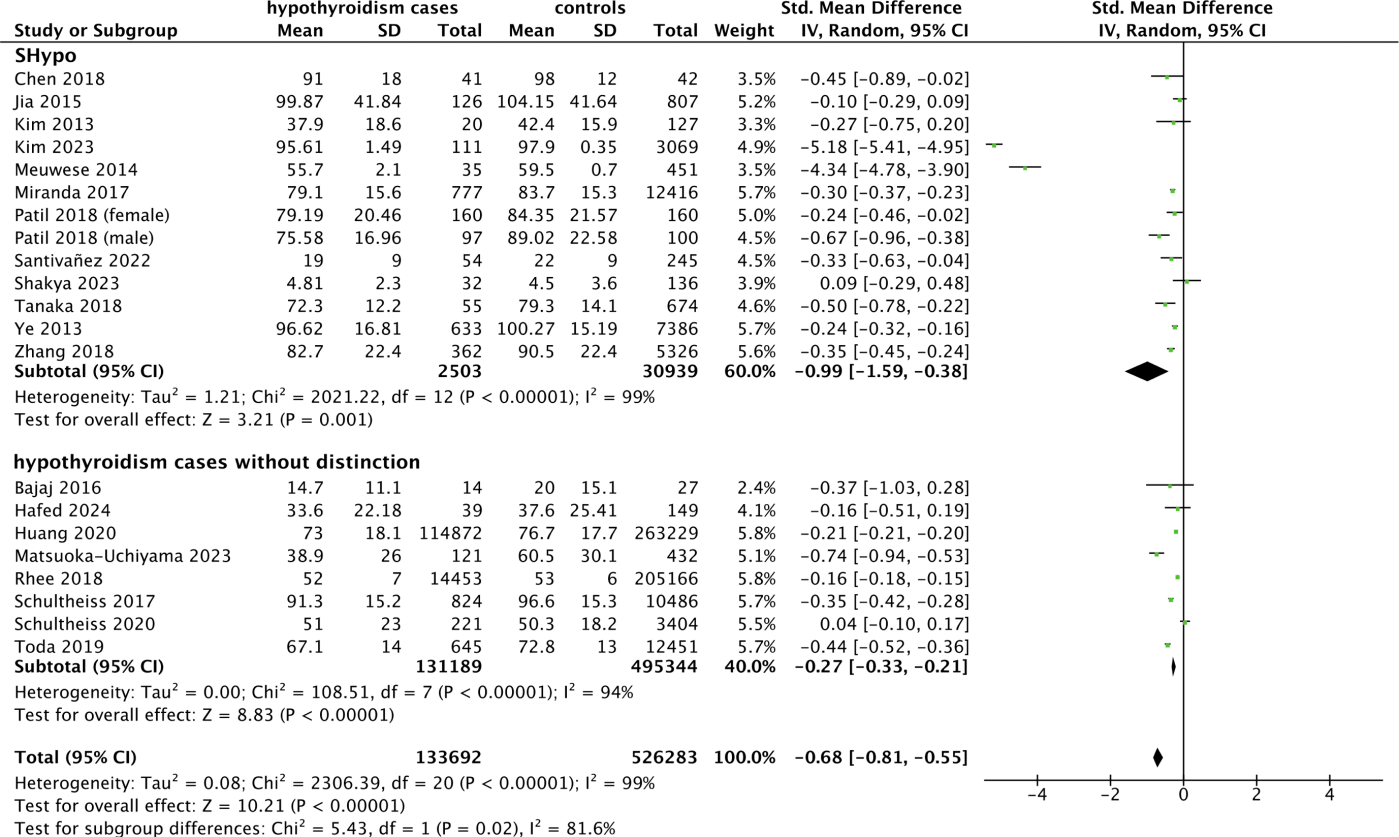
Forest plot of the association between hypothyroidism and CKD prevalence. *CKD, chronic kidney disease*.

As shown in **Figure 2**, pooled hypothyroidism was associated with a higher risk of CKD (OR 1.94, 95% CI [1.62, 2.32], P < 0.00001). Subgroup analysis revealed that SHypo and non-distinct hypothyroidism were positively associated with CKD (OR 1.87, 95% CI [1.55, 2.27], P < 0.0001; OR 1.98, 95% CI [1.36, 2.89], P = 0.0004, respectively).

As shown in **Supplementary Figure 1**, overall, hypothyroidism was associated with a higher risk of ESRD (OR 1.92, 95% CI [1.23, 2.99], P = 0.004). Subgroup analysis was conducted based on the presence of prior kidney dysfunction, and the results suggested that hypothyroidism remained significantly associated with ESRD in the general population (OR 3.16, 95% CI [1.58, 6.35], P = 0.001). However, no evidence of an association between hypothyroidism and ESRD was found among participants who already had CKD.

### Association between hypothyroidism and the incidence of CKD

Three cohort studies included 4,042 patients with varying severities of hypothyroidism. As shown in **Supplementary Figure 2**, no significant association was found between hypothyroidism and the CKD incidence.

### Associations between hypothyroidism and the biochemical parameters of kidney function

Nine case-control studies, which included 936 patients, compared serum urea levels between patients with hypothyroidism and controls (**Supplementary Figure 3**). Overall, hypothyroidism was associated with elevated serum urea levels according to pooled data from the two subgroups (SMD 0.47, 95% CI [0.01, 0.93], P = 0.04), whereas SHypo was not significantly associated with serum urea levels.

Given the variability in baseline kidney function across the populations in the above analysis, studies that were specifically conducted in patients with underlying kidney dysfunction were removed from the sensitivity analysis. As shown in **Supplementary Figure 4**, the above conclusions remained robust. Pooled hypothyroidism was significantly associated with elevated serum urea levels (SMD 0.74, 95% CI [0.08, 1.39], P = 0.03), whereas SHypo was not.

A total of 13 case-control studies were included in the analysis of the association between hypothyroidism and serum creatinine levels (**Supplementary Figure 5**). Among them, seven and six studies covered patients with SHypo and hypothyroidism without distinction, respectively. Similar to the findings for serum urea levels, no significant association was found between SHypo and serum creatinine levels. However, we verified a significant association between pooled hypothyroidism and serum creatinine levels (SMD 0.61, 95% CI [0.20, 1.02], P = 0.004).

We further analyzed the association between hypothyroidism and serum creatinine levels after excluding studies specifically conducted in patients with underlying kidney dysfunction. As shown in **Supplementary Figure 6**, pooled hypothyroidism was still significantly associated with serum creatinine levels (SMD 1.14, 95% CI [0.44, 1.83], P = 0.001). Moreover, SHypo was positively associated with serum creatinine levels (SMD 1.55, 95% CI [0.32, 2.78], P = 0.01).

### Association between hypothyroidism and the eGFR

**Figure 3:**
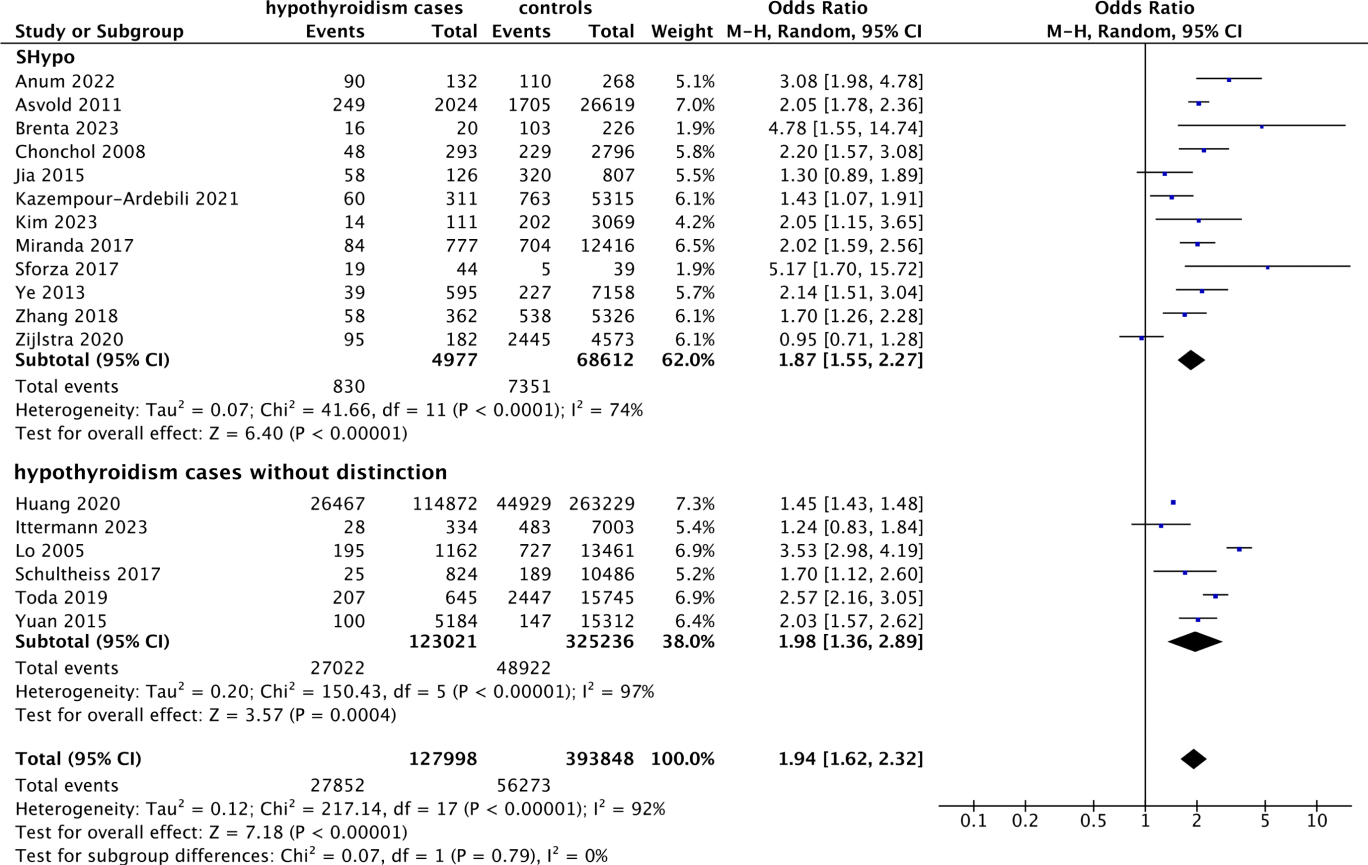
Forest plot of the association between hypothyroidism and eGFR. *eGFR, estimated glomerular filtration rate*.

As shown in **Figure 3**, the cross-sectional association between hypothyroidism and the eGFR was investigated in 20 studies. Twelve and eight studies were conducted in patients with SHypo and hypothyroidism without distinction, respectively. There was a significant association between hypothyroidism and a decreased eGFR (SMD -0.68, 95% CI [-0.81, -0.55], P < 0.00001) if all the hypothyroidism cases were pooled. Subgroup analysis suggested that SHypo and hypothyroidism without distinction were also significantly associated with a decreased eGFR (SMD -0.99, 95% CI [-1.59, -0.38], P = 0.001 and SMD -0.27, 95% CI [-0.33, -0.21], P < 0.00001, respectively).

Studies specifically conducted in patients with prior kidney dysfunction were removed in the sensitivity analysis. As shown in **Supplementary Figure 7**, the relationships between SHypo, non-distinct hypothyroidism, and pooled hypothyroidism and the eGFR still reached significance (SMD -1.31, 95% CI [-2.07, -0.56], P = 0.0006; SMD -0.33, 95% CI [-0.47, -0.18], P < 0.0001; SMD -1.04, 95% CI [-1.37, -0.70], P < 0.00001, respectively).

### Effects of LT4 replacement on kidney outcomes in hypothyroidism patients

Serum creatinine levels were assessed in six studies. As shown in **Supplementary Figure 8**, overall, serum creatinine levels in hypothyroidism patients decreased significantly after LT4 replacement (SMD -0.85, 95% CI [-1.53, -0.17], P = 0.01). However, serum creatinine levels in SHypo patients did not significantly differ from baseline.

Three and eight studies analyzed changes in the eGFR after LT4 treatment in patients with SHypo only and hypothyroidism without distinction, respectively. As shown in **Supplementary Figure 9**, we did not find an explicit effect of LT4 replacement on the eGFR in pooled hypothyroidism patients.

## Discussion

To our knowledge, no meta-analysis investigating the association of hypothyroidism and specific kidney outcomes have been published so far, thus this study would be of high clinical significance. This study verified that hypothyroidism (including SHypo) was significantly associated with the prevalence of CKD and reduced eGFR. Furthermore, hypothyroidism was also significantly cross-sectionally associated with increased serum urea and serum creatinine. These conclusions are consistent with our previous experience in clinical practice. However, this study concluded that hypothyroidism was not associated with the incidence of CKD or the progression of ESRD in subjects with prior CKD. LT4 treatment was not associated with improvement in eGFR, either in non-distinct hypothyroidism or in SHypo patients.

Thyroid dysfunction cannot be diagnosed or treated roughly based on the reference interval provided by the test kit. TSH measurement ​​is affected by various factors including age, BMI, pregnancy, etc (65). Additionally, comorbidities such as CKD, cardiovascular disease and osteoporosis should also be fully considered when treating hypothyroidism in clinical practice. Assessment of kidney function is necessary. Regarding the specific practice paradigm, published guidelines within the endocrinology community are not fully consistent on this issue (66–70). The pathophysiology of kidney dysfunction in hypothyroidism patients may attribute to three factors (71–73). First, low thyroid hormone levels can inhibit the development of glomeruli and renal tubules, and TSH receptors can be significantly expressed in the kidney. Second, hypothyroidism can induce altered tubular ion channel activity and increased glomerular capillary permeability to proteins. Third, hemodynamic disorders can indirectly affect the eGFR because the effective circulating blood volume and vascular resistance increase with hypothyroid status. CKD (especially ESRD) is often accompanied by decreased thyroid hormone levels in clinical practice, which is consistent with nonthyroidal illness. In the absence of primary thyroid dysfunction, its pathogenesis may be related to peripheral deiodinase inactivation and decreased central sensitivity to thyroid hormone (74, 75). We hereby provide more evidence for the summary of previous clinical experience. Hypothyroidism and SHypo are both significantly cross-sectionally associated with CKD as well as a decreased eGFR.

No significant association between hypothyroidism and the CKD incidence was observed in this study. Moreover, the notable association between hypothyroidism and ESRD disappeared once studies, especially those including participants with preexisting kidney dysfunction were excluded. The results of the present study also revealed no significant improvement in the eGFR after LT4 replacement. Meuwese et al. conducted an IPD meta-analysis based on several large cohorts of the Thyroid Studies Collaboration and reported similar findings. Hypothyroidism patients did not demonstrate a significant reduction in kidney function during follow-up. Researchers speculate that the positive cross-sectional results cannot completely rule out that kidney failure causes changes in thyroid hormone levels (76). More large, real-world prospective studies are needed to further explore the exact association between the two variables.

In addition to its association with the eGFR, hypothyroidism was significantly associated with elevated serum urea and serum creatinine. However, SHypo appeared to have a less robust association with these biochemical indicators. In the overall population, SHypo was not significantly associated with serum creatinine levels. Analysis of SHypo patients with a history of kidney dysfunction also revealed that LT4 replacement did not improve serum creatinine levels, which partially confirms the above results. We speculate that the association between SHypo and serum creatinine should be examined separately in different populations and that there might be a borderline association between them. After studies particularly in those with the presence of kidney dysfunction were excluded, a significant association between SHypo and serum creatinine levels was observed, and LT4 treatment was also effective in studies \without the presence of prior kidney dysfunction. We speculate that the inconsistency in the above results may be due to non-kidney-related factors of the creatinine measurements. Hypothyroidism itself might induce a reduction in the serum creatinine measurements through pathophysiological processes such as reduced muscle metabolism and fluid retention (77). In SHypo patients combining with a personal history of kidney dysfunction, the opposing effects of the above pathophysiological processes and primary kidney diseases may result in less significant changes in serum creatinine levels, thereby inducing non-significant results. These findings also suggest that we should comprehensively evaluate multiple indicators of kidney function report in clinical practice. The pathophysiological explanation for the relationship between SHypo and serum creatinine levels remains speculative and incomplete. The number of included studies is relatively small in this set of analysis. Future studies with larger cohorts and basic research are required to rationally elucidate the relationship between SHypo and serum creatinine, as well as the necessity and timing of initiation of LT4 replacement.

The limitations of this study are described below. First, because the sample sizes in the studies were generally small, we could not perform subgroup analyses based on age, sex, the TSH threshold or the follow-up duration. The results may be biased due to lack of adjustment for comorbidities that may affect kidney function. Second, some kidney-related indicators were not included in this study because of the lack of evidence. Third, we could not accurately assess kidney structural or functional abnormalities beyond three months because of the practical limitations of epidemiological investigations. In previous studies, CKD was diagnosed according to the eGFR via a single measurement, which may have led to discrepancies in the true prevalence. Fourth, although the sensitivity analysis suggested relatively robust results, the differences in the weights of several specific studies are of concern. Fifth, the diagnostic criteria of hypothyroidism and the measurements or formulas of kidney indicators were inconsistent across the various studies; thus, this heterogeneity may have resulted in a certain bias in the conclusions. Briefly, the results of the sensitivity and heterogeneity analyses are bound to suggest a certain risk of bias, which is unavoidable in meta-analyses. The clinical significance of this study needs to be confirmed by more large-sample cohort studies and RCTs.

## Conclusion

This study conducted a systematic review and meta-analysis of studies on hypothyroidism and kidney outcomes based on evidence from the past two decades. Both non-distinct hypothyroidism and SHypo were found to be cross-sectionally associated with CKD and a reduced eGFR. However, hypothyroidism did not appear to be significantly associated with the incidence or further progression of CKD. No significant increase in eGFR was observed after LT4 replacement was given to patients with non-distinct hypothyroidism or SHypo to restore euthyroidism. Further consideration and exploration of the bidirectional relationships between hypothyroidism and kidney outcomes are warranted, as this will have substantial benefits for the management of hypothyroidism and CKD.

## Data Availability

The original contributions presented in the study are included in the article/**Supplementary Material**. Further inquiries can be directed to the corresponding author.
