# Supplementary figures and images for: Association between hypothyroidism, levothyroxine replacement and kidney function outcomes: a systematic review and meta-analysis
Source: Front Endocrinol (Lausanne). 2026 May 1;17:1841255. doi: 10.3389/fendo.2026.1841255 (PMC13175885; doi:10.3389/fendo.2026.1841255)

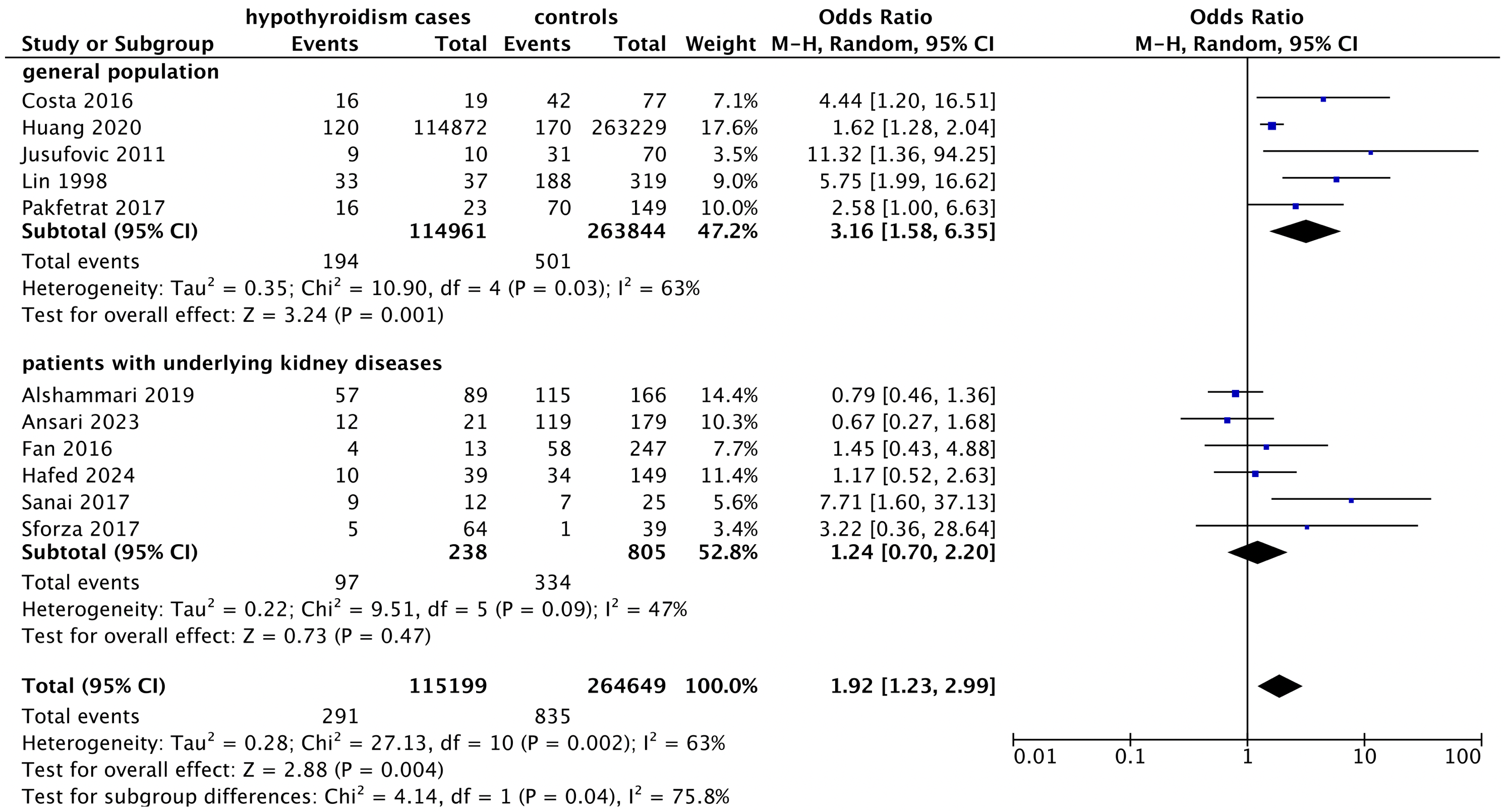

Supplement: Supplementary file 2 [file Image1.tiff]

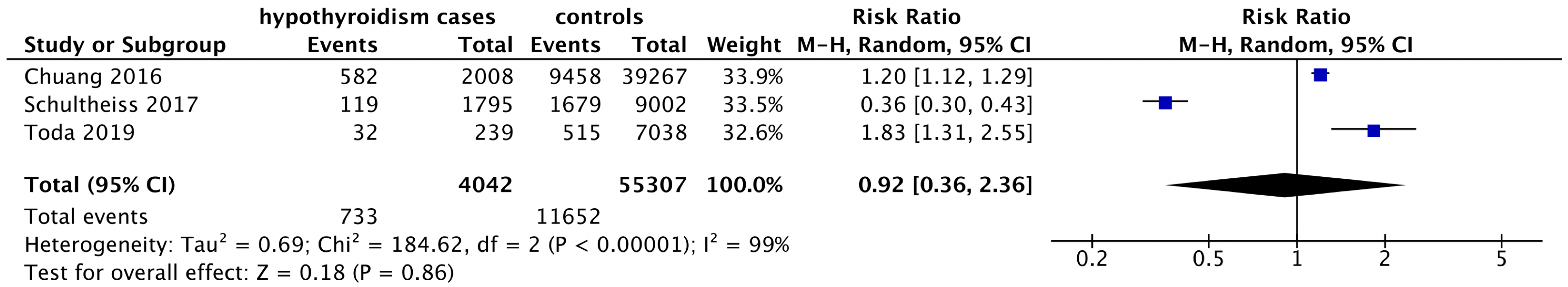

Supplement: Supplementary file 3 [file Image2.tiff]

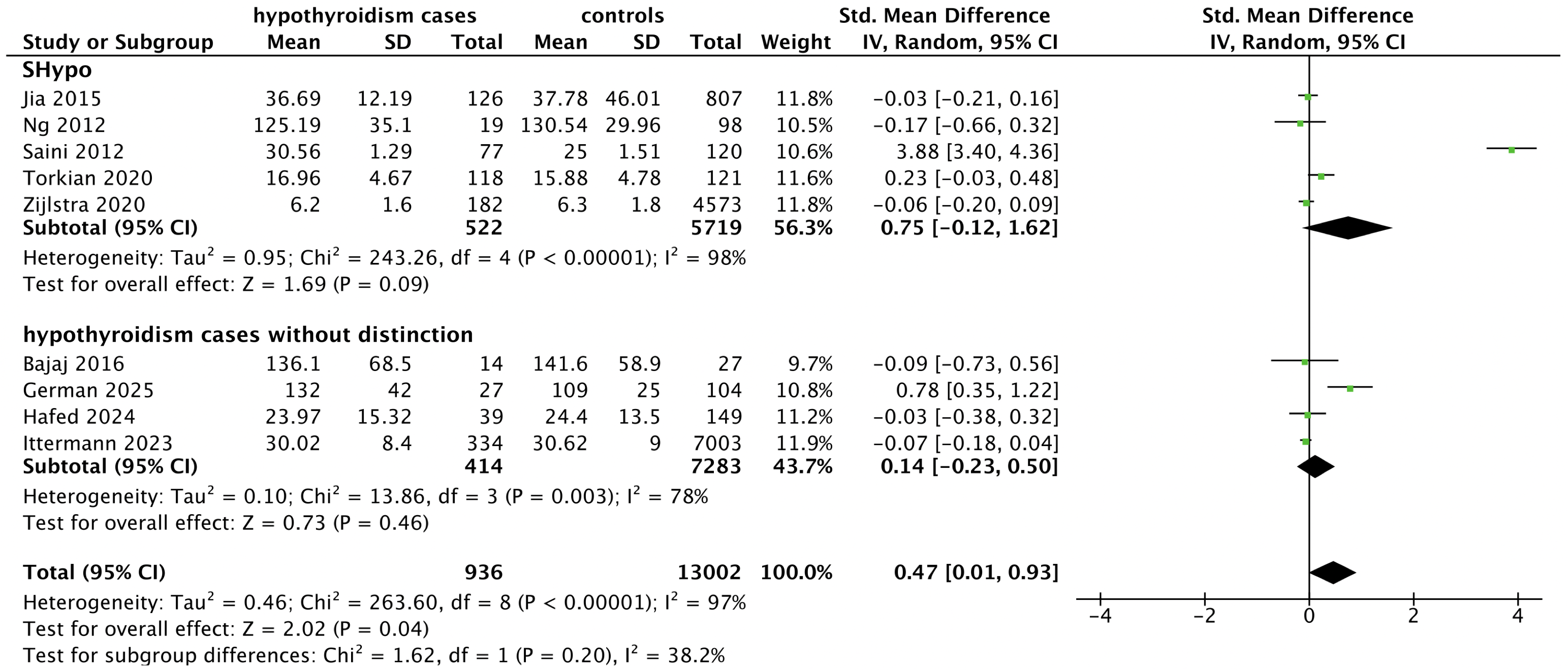

Supplement: Supplementary file 4 [file Image3.tiff]

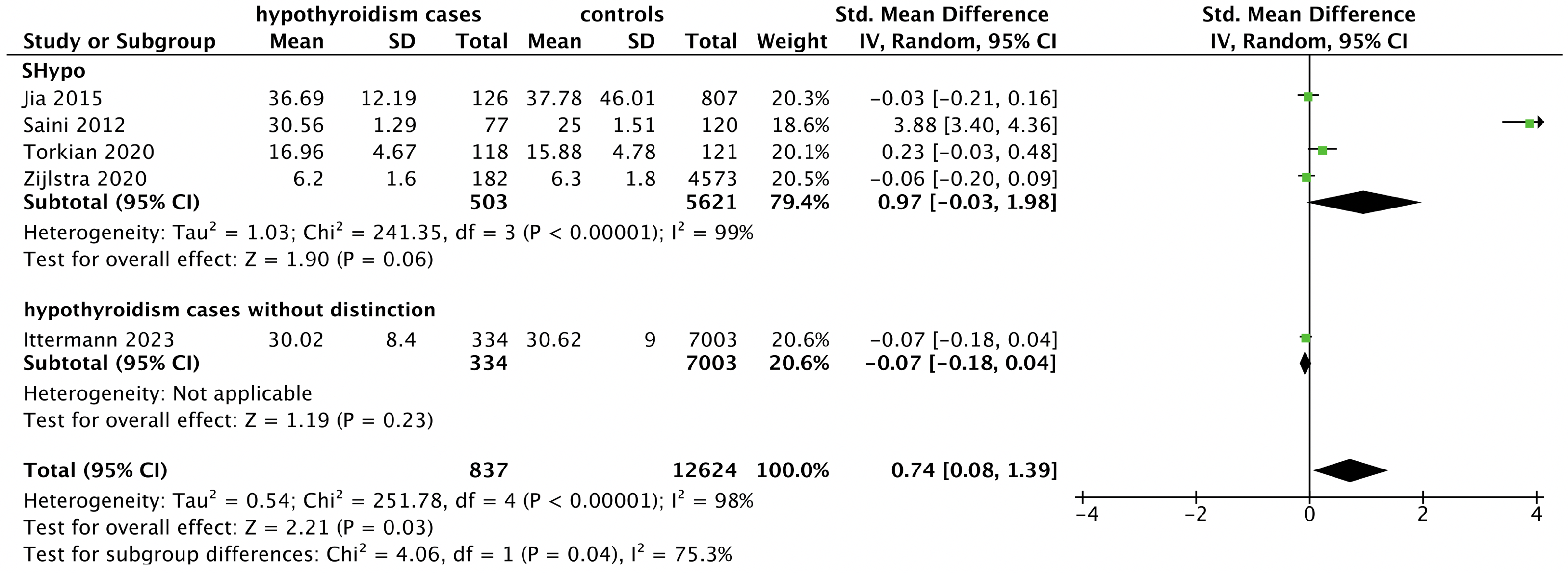

Supplement: Supplementary file 5 [file Image4.tiff]

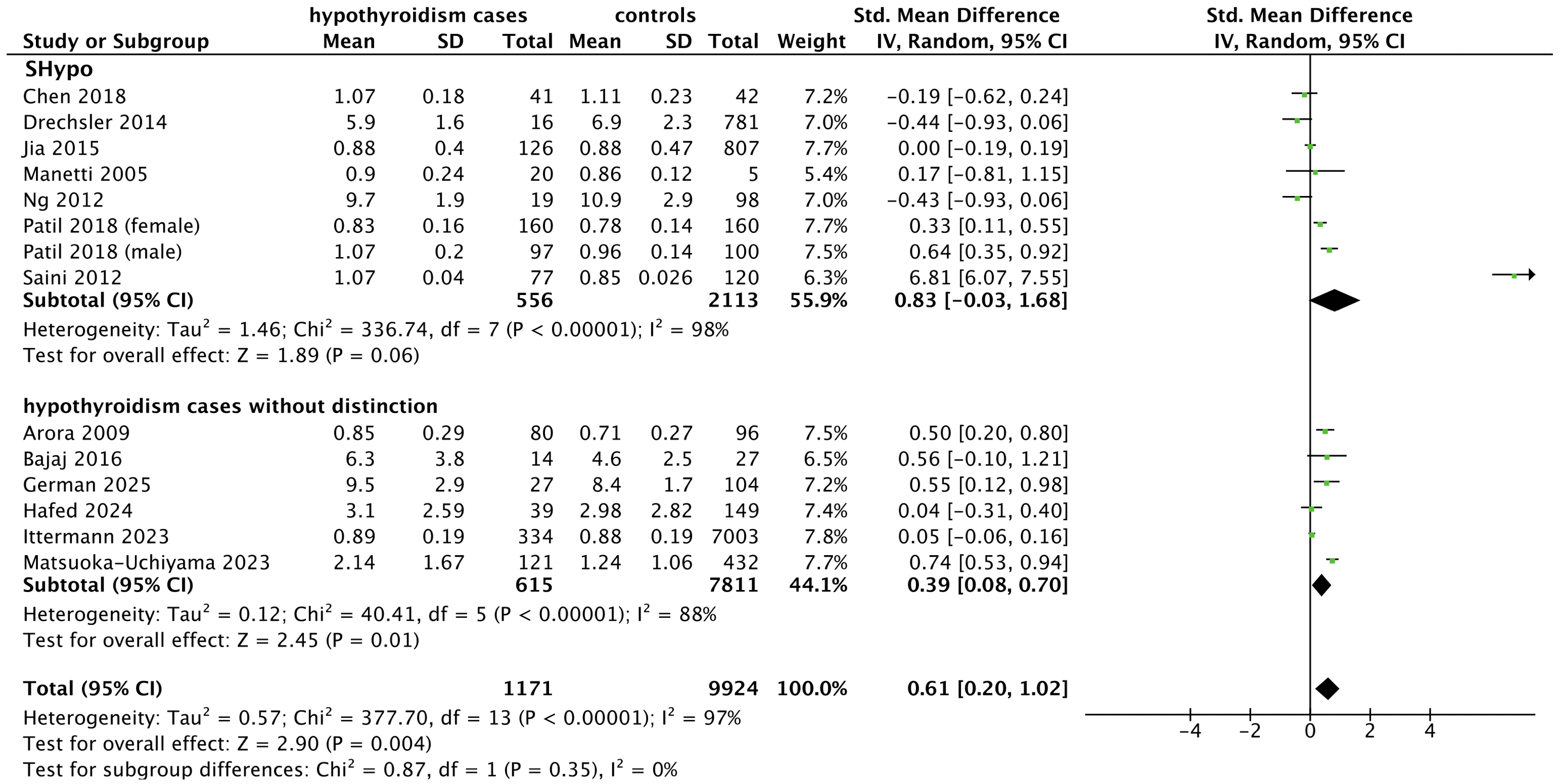

Supplement: Supplementary file 6 [file Image5.tiff]

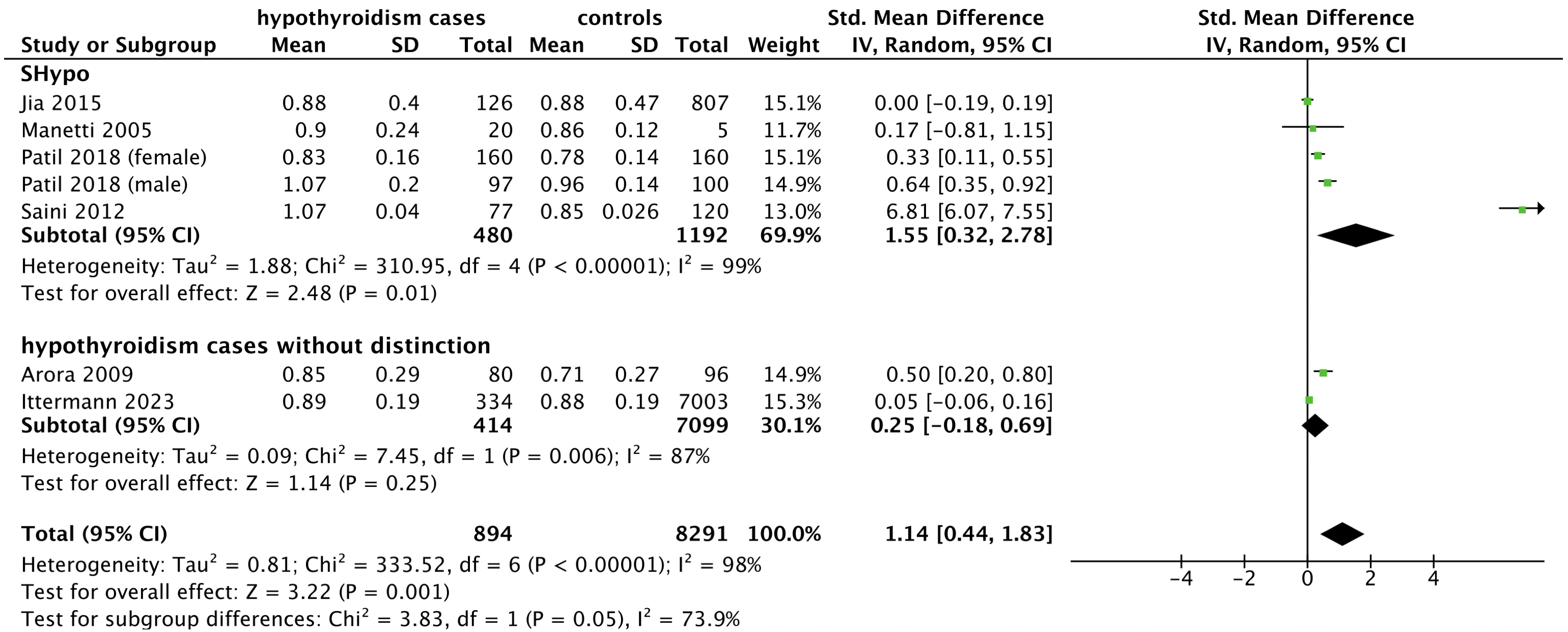

Supplement: Supplementary file 7 [file Image6.tiff]

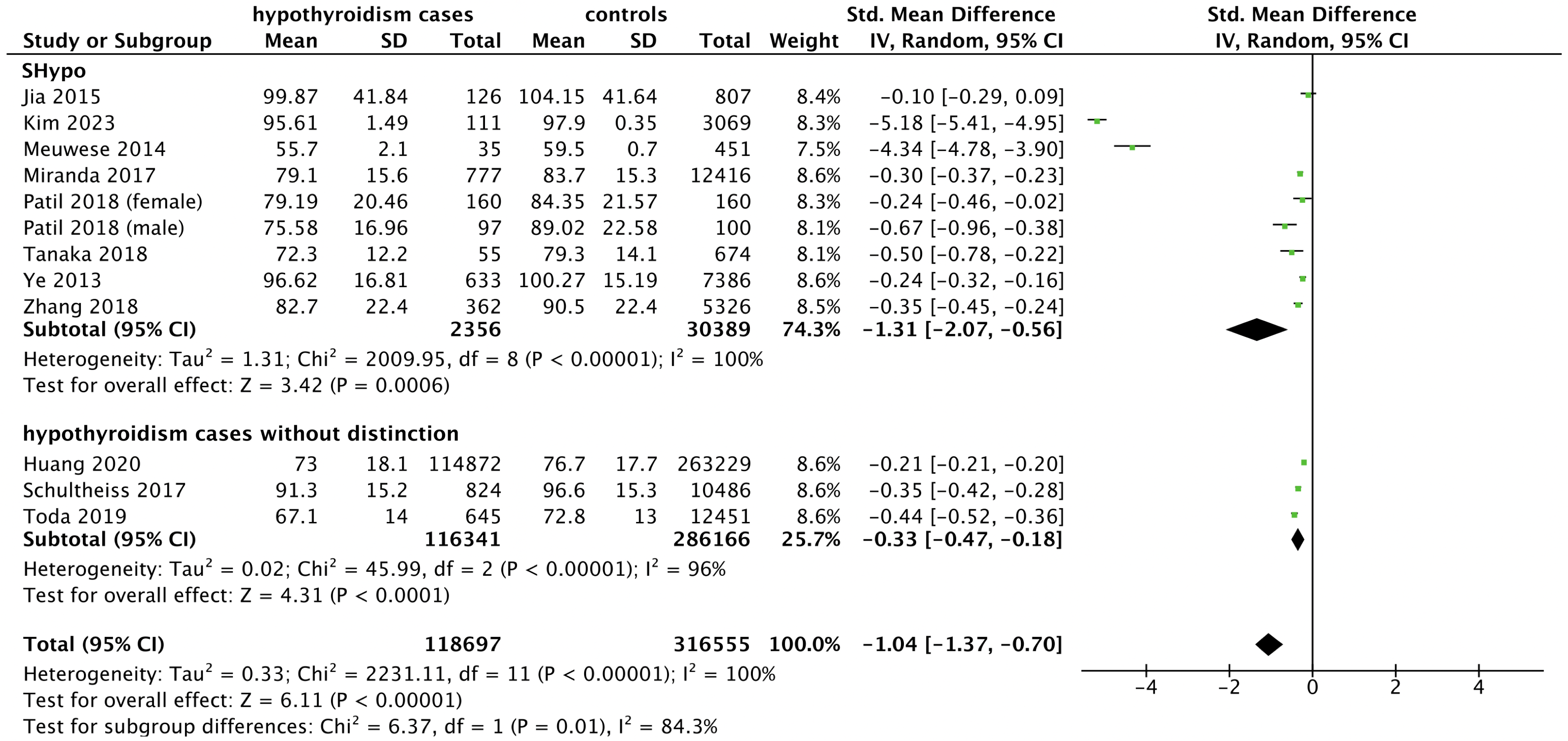

Supplement: Supplementary file 8 [file Image7.tiff]

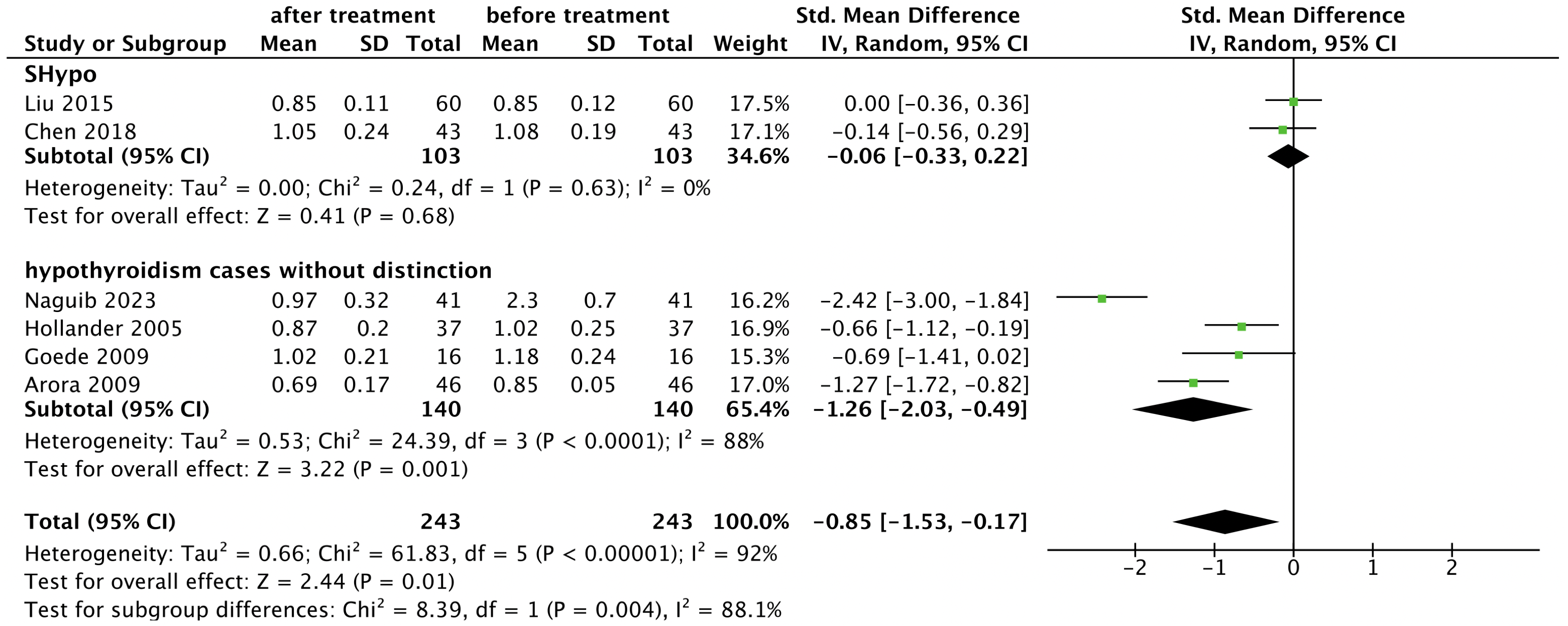

Supplement: Supplementary file 9 [file Image8.tiff]

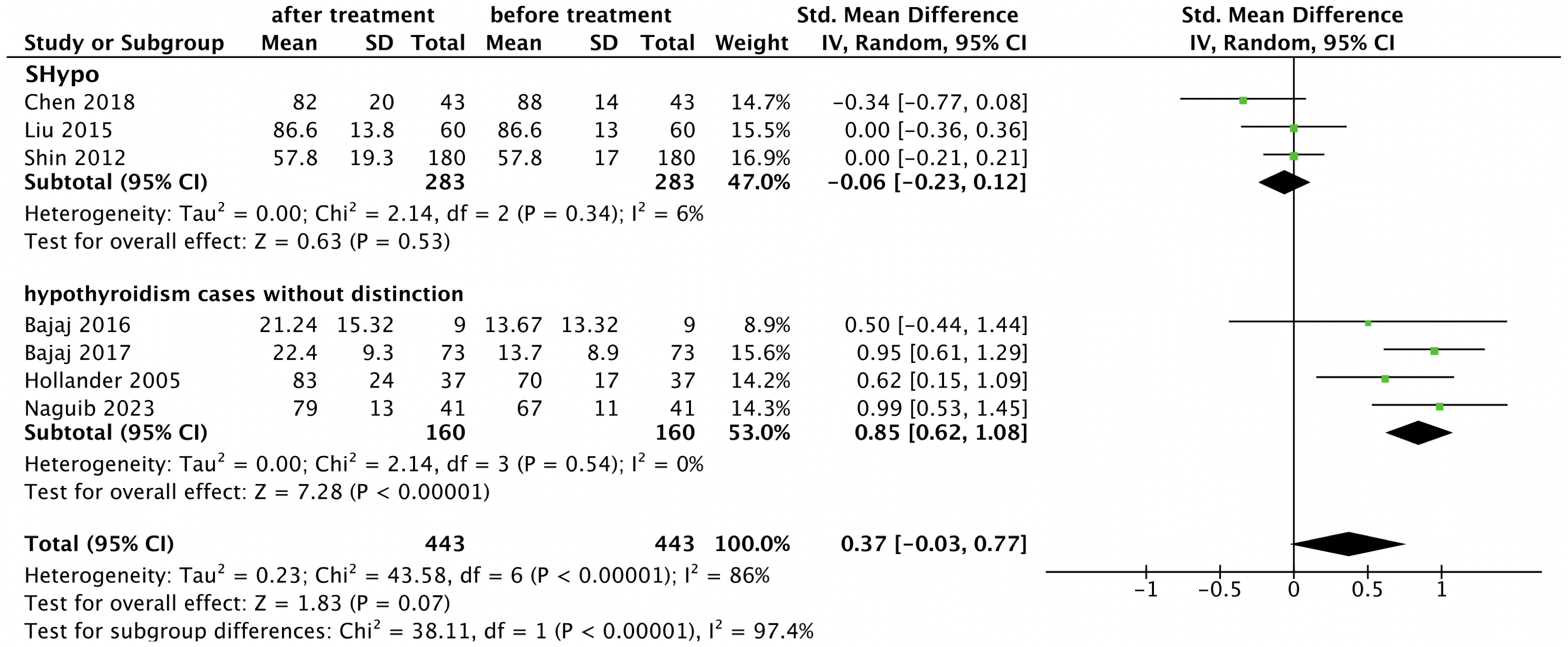

Supplement: Supplementary file 10 [file Image9.tiff]
